# Air in Wounds

**Published:** 1870-09

**Authors:** 


### Air in Wounds.

Mr. Skey expresses very reasonable doubts of the injurious influence of atmospheric air in wounds. In the case of compound fractures, he attributes the slowness of the healing process, and other untoward symptoms, rather to the laceration and contusion of the structures than to the admission of air ; adding that in operations for empyema and hydrothorax, he has never made any attempt to exclude air, and quoting one case in which he and the late Dr. Todd intentionally admitted air enough to take the place of six pints of serous fluid, without the slightest evil result.—*Medical Gazette.*

